# Coating Methods for Surface Modification of Ammonium Nitrate: A Mini-Review

**DOI:** 10.3390/ma9070502

**Published:** 2016-06-23

**Authors:** Baha I. Elzaki, Yue Jun Zhang

**Affiliations:** 1 School of Chemical Engineering, Nanjing University of Science and Technology, Nanjing 210094, Jiangsu, China; bhaeldeen8888@yahoo.com; 2 Department of Chemical Engineering, College of Engineering, Karary University, Omdurman 12304, Sudan

**Keywords:** ammonium nitrate, chemical coating, encapsulation coating, hygroscopicity, modification surface, physical coating

## Abstract

Using ammonium nitrate (AN) as a propellant oxidizer is limited due to its hygroscopicity. This review consolidated the available information of various issues pertaining to the coating methods of the surface modification of ammonium nitrate for reducing its hygroscopicity. Moreover this review summarizes the recent advances and issues involved in ammonium nitrate surface modification by physical, chemical and encapsulation coating methods to reduce the hygroscopicity. Furthermore, coating materials, process conditions, and the hygroscopicity test conditions are extensively discussed along, with summaries of the advantages and disadvantages of each coating method. Our findings indicated that the investigation and development of anti-hygroscopicity of AN, and the mechanisms of surface modification by coating urgently require further research in order to further reduce the hygroscopicity. Therefore, this review is useful to researchers concerned with the improvement of ammonium salts’ anti-hygroscopicity.

## 1. Introduction

**Figure 1:**
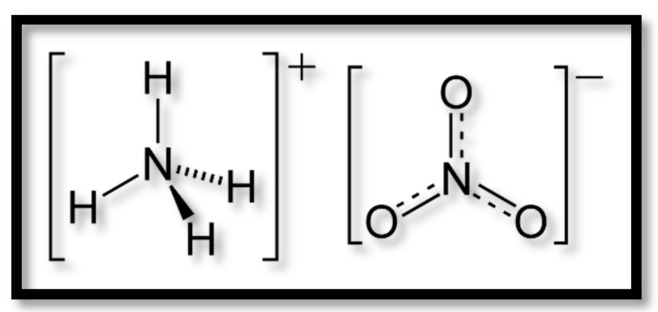
Formula of ammonium nitrate.

Ammonium nitrate (AN) NH_4_NO_3_ is an inorganic salt (Figure 1). It is low cost, safe and has a simple manufacturing process [1,2].

**Figure 2:**
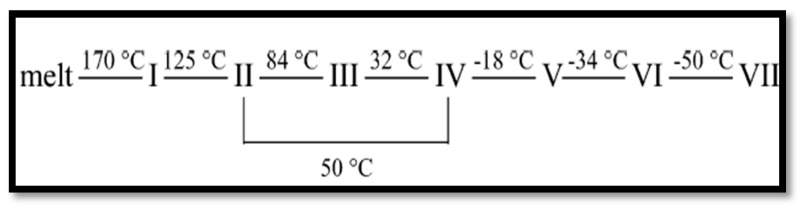
Phase transitions of AN.

Pure AN is a colorless salt with relative density equal to 1.725 g/cm^3^ and a melting point of 169 °C. The decomposition process is significantly promoted at a slightly higher temperature of 210 °C [3,4,5]. AN is widely used in fertilizers, and as an active component in explosive mixtures. It is a strong oxidizer and an insensitive explosive [4,6,7]. AN has a positive oxygen balance, and the nitrate ion acts as the source of oxygen, resulting in a promising compound as a propellant and gas generator [8,9,10]. The phase transition in pure AN can also occur at different temperatures as demonstrated in Figure 2 [11,12].

Recently, there has been renewed interest in a clean-burning propellant, aiming at green propellants with HCl-free burning. Ammonium perchlorate (AP) was replaced by different oxidizers because it releases toxic gases. There are ammonium dinitramide (ADN) and hydrazinium nitroformate (HNF) energetic oxidizers, but these are not available in adequate quantities [13,14,15,16]. AN-based composite propellants are primarily attractive due to the combustion products without toxic gases (HCl-free) [17]. Although ammonium nitrate is suitable as an oxidizer in large rocket motors, it is, however, restricted due to the hygroscopicity [18,19]. Furthermore, the surfaces of the AN solid particles are strongly polar, which cause hygroscopicity [20,21]. Hygroscopicity of AN has been pinpointed as the major cause for caking, and has become the biggest obstacle for its usage explosives and propellants [22,23]. Generally, reducing the hygroscopicity of AN has been studied by many researchers using surface modification techniques, by coating AN particles with different types of coating methods, in particular physical, chemical and encapsulation coating methods. To the best of our knowledge, the available coating methods that modify the surface of AN have not been aggregated and thoroughly reviewed. Therefore, the objective of this paper is to review the recent studies for the surface modification of AN to reduce the hygroscopicity, with an attempt to summarize the relevant information with emphasis on the main coating methods such as physical coating, chemical coating, and encapsulation coating methods. This consequently increases the effectiveness of the use of AN as a solid propellant oxidizer. The above-mentioned methods and concerns have been deeply discussed and compared to each other. The recent advances and development in this regard have been thoroughly addressed.

## 2. Coating Methods

Surface modification of AN with several coating methods was applied in order to reduce hygroscopicity, which has been tackled by several previous studies. This section will discuss these methods as follows: 

### 2.1. Physical Coating Method

**Figure 3:**
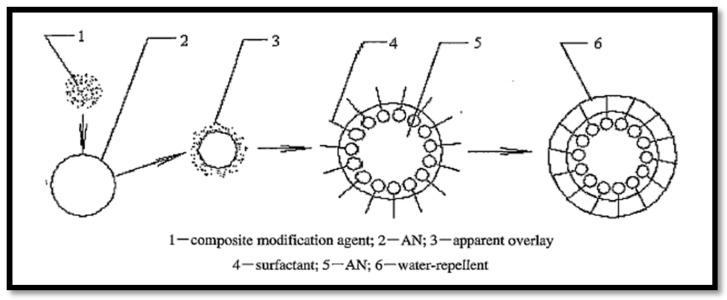
Micro-structure of composite-modified water-resistant powdery AN.

The physical coating method is used to modify the surface of AN particles to reduce the hygroscopicity. In this method the interaction between the surface of AN particles and the coating agents occurs by physical absorption; for example, the polar head of the surfactant has an affinity to the polar surface of AN particles and makes a hydrophobic uniform membrane on the surface with the hydrophobic tails of the surfactant materials [24]. In addition, the success of this process mainly depends on the solubility (the solubility of the surfactant is controlled by the temperature) of the surfactant and the insolubility of AN, as well as on the suspension of particles in aqueous solution, because the soluble surfactant can easily deposit on insoluble particles of AN, with the suspension making a uniform coating layer on the surface of the particles [25]. Moreover, the stirring and densities of solvents have significant roles in making the suspension system [26]. In fact, the mechanism of coating by surfactant materials probably varies from surfactant group to surfactant group and even within the same surfactant groups due to the different lengths of the tails [27]. In 1996, Chattopadhyay used stearic acid to coat the surface of the AN particles, and hydrophobic film was formed to achieve the purpose of preventing moisture. This study provided a worthy explanation on coating AN with stearic acid; however, no details were mentioned about the coating process and the hygroscopic test conditions [28]. Furthermore, there was a water-resistant base of an AN explosive composition made by blending the particles of AN with different groups, such as alkaline earth metals and earth metal salts of aliphatic carboxylic acids [29]. AN particles were modified by a composite consisting of anion, cationic, non-ion, and amphoteric surfactants. Therefore, the dispersion improved by adding a quantity of a dispersant agent such as diatomate or vermiculite; in addition the anti-water mechanism is shown in Figure 3. To our knowledge this is the first study on the coating of AN with a composite surfactant, which can be used as the base for the surface modification of AN with composite surfactant [30].

Another piece of research showed that the AN particles were coated with mineral oil. Under a temperature of 20 °C, 65% relative humidity, and the absorption moisture time of 96 h, the decline of the moisture absorption rate increased to 65% with the mass ratio of the coating layer at 1%. It is worth mentioning that this study was classified as the highest level in the literature for the current rate of decline in the physical coating for a decrease in moisture [31]. Furthermore, Kun-lun Hu et al. used stearic acid, resin, wax, etc., as coating materials; the particle sizes of AN were 20–40 mesh in the melting process. Under the conditions of a temperature at 25 °C, a relative humidity of 90%, and an absorption moisture time of 8 h, the mass ratio of the coating layer was decreased from 6.25% to 2.17%, and the decline was 65.28%. This study used a short duration of the absorption moisture test with a high mass ratio of the coating layer [32]. Moreover, the coupling cationic surfactant (*N*,*N*-Dimethyldodecylamine) has been used to improve the anti-hygroscopicity, anti-caking and explosion performance of AN particles [33]. For reducing hygroscopicity the heating melt method with different amounts of paraffin was used, resulting in a modification of AN particles [34]. Octadecylamine was also used to modify ammonium nitrate and characterized the contact angle to investigate the explosive performance [35]. The mechanism of interaction between ammonium nitrate and octadecylamine was studied by using SEM, XRD, XPS and IR, which resulted in the coating of the ammonium nitrate surface with octadecylamine through physical absorption, which improved the explosion performance [36]. The performances of modified AN using physical coating methods are summarized in Table 1.

### 2.2. Chemical Coating Method

The chemical coating is a method used for AN particle modification to reduce hygroscopicity. In this method the adsorption of coating materials on the surface of the AN particles causes a chemical reaction resulting in the formation of a coating layer on the particles. AN particles with a size of 60–80 mesh were coated by silane coupling agents KH550 and KM602; the hygroscopicity test conditions were a temperature of 35 °C, a relative humidity of 92%, and a time of 24 h, and the decline of absorbed moisture was 38.7% in the AN modified with KH550. However, the mass ratio of the coating layer was not determined [37]. In contrast the decline of the moisture absorption increased to 56.25% when the AN particles were coated with an anionic surfactant mixture of lignosulfonate and urea, at a temperature of 35 °C, a relative humidity of 55%, and a moisture absorption time of 24 h. However, the mass ratio of the coating layer was not mentioned, nor was the relative humidity near the AN critical relative humidity [38]. Another piece of research showed the modification of AN particles sized 50–75 mesh with silane coupling agent KH792 as the coating material. The hygroscopicity was tested under conditions of a temperature of 20 °C, a relative humidity of 88%, and a moisture absorption time of 8 h, and the absorption rate of the moisture was decreased from 12.86% to 10.92%, with a decline of 15.08%. In this study, the mass ratio of the coating layer was not determined, and the decline of the moisture absorption was low [39]. Table 2 shows the performance of modified AN using a chemical coating method.

### 2.3. Encapsulation Methods

**Figure 4:**
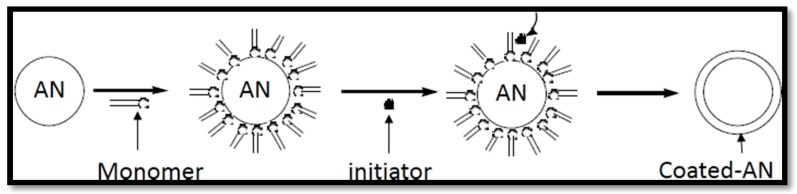
Monomer and polymeric particles adsorbed surface-coating schematic.

Encapsulation is a new coating method. The capsule-like coating layer is formed on the surface of AN particles, which are homogeneous and of a certain thickness. The mechanism of encapsulation is shown in Figure 4. The process of encapsulation coating involves dispersing the AN particles in a solution of an encapsulating material dissolved in an organic solvent, suspending the whole system, and then evaporating away the organic solvent [40,41]. Based on the reported literature, the materials forming capsules are divided into three categories:

(1) Cellulose derivatives, such as nitrocellulose, ethyl cellulose.
(2) Resin and plastics, such as poly methyl methacrylate, acrylic acid resin, polyvinyl acetylate, polystyrene, etc.
(3) Elastomeric polymer such as polyurethane elastomer.

Depending on the different processes and approaches of the encapsulation coating process, there are three major categories:

(1) Mechanical and physical methods such as the spray-drying method.
(2) Physical methods such as the phase separation method.
(3) Chemical methods such as the precipitation polymerization method.

Many studies were done to deal with the anti-moisture of ammonium nitrate. The study showed the modification of AN particles by the polymerization action in the interface of xylene/AN; the odctadecylamine forms a monolayer with self-assembling through hydrogen bonding on the surface of AN particles [42]. Meanwhile, the structure of the self-assembled monolayer film on the surface modified with octadecylamine and dimethyl benzene was characterized. This study has an explanation of the interaction between the monolayer and the AN [43]. The microencapsulation technology has been used for reducing the hygroscopicity and agglomeration of AN by coating it with polystyrene (PS) [44]. The AN particles were modified to improve the hygroscopicity and caking by coating particles using encapsulation through a physico-chemical process technique [45]. In addition, the AN particles were modified by liquid phase separation techniques using polyvinyl butyral (PVB), in order to reduce the hygroscopicity and caking capability [46]. The AN particles were modified by liquid phase separation technology, using polyvinyl acetate (PVAC), polyacrylamide (PAM), and polyethylene glycol (PEG) as coating materials. In this study, the modification effects of PVAC and PAM in the AN decreased the hygroscopicity and the coated particles, which have a better spherical shape [47]. Moreover, Yue (2003) used 10% methyl methacrylate monomer as a coating material and 0.7% azobisisobutyeonitrile as an initiator in the reaction medium of petroleum ether, cyclohexane and toluene, with a reaction time of 8 h at 60 °C. Under the conditions, at temperature of 32 °C, a relative humidity of 90% and an absorption moisture time of 24 h, the absorption rate of moisture was decreased from 12.80% to 9.91%, and the decline was 22.50%. The absorption moisture test conditions were good, but the mass ratio of the coating layer and the AN particle sizes were not determined [37]. The surface polarity of AN was decreased by using a spray-drying technique, and a surfactant, a coupling agent and polyvinyl butyral as coating materials. In this study, the duration of the hygroscopicity test was short [48]. Furthermore, AN was coated with 4% polystyrene monomer, and 1% azobisisobutyeonitrile as an initiator was used in the polymerization reaction medium chloroform. Under the conditions of a temperature of 20 °C, a relative humidity of 75%, and an absorption moisture time of 12 h, the mass ratio of the coating layer was decreased from 1.24% to 0.623%, and the decline was 88.8%. The reported hygroscopicity was also affected by the particle size of the uncoated AN [49]. In 2013, Zhicheng et al. used methyl methacrylate as a monomer in precipitation polymerization, and azobisisobutyeonitrile as an initiator, and chloroform as a solvent, and the reaction time was 8 h at 65 °C. Under the conditions of a temperature of 35 °C, a relative humidity of 92%, and an absorption moisture time of 24 h, the absorption rate of the moisture was decreased from 12.26% to 8.78%, the decline was 28.35%, and the mass ratio of the coating layer was 0.48, which is the highest level in literature reported until now. In this study, the absorption moisture test conditions were high [50]. The performance of modified AN using encapsulation coating is summarized in Table 3. Although the surface modification of AN using physical coating, chemical coating and encapsulation coating methods had advantages in reducing the hygroscopicity, these methods also had disadvantages as shown in Table 4.

## 3. Conclusions

The hygroscopicity of AN has restricted its application in explosives and propellants. This mini-review clearly summarized the surface modification of AN particles using physical, chemical and encapsulation coating methods to reasonably reduce the hygroscopicity. Our findings indicated that further exploration and improvement of the anti-hygroscopicity of AN and the mechanisms of surface modifications are urgently required for a greater reduction in hygroscopicity.

## Figures and Tables

**Table 1:** Performance of coated AN using the physical coating method.

No.	Year	Absorption Moisture Rate (%)	Mass Ratio of Coating Layer (%)	Absorption Moisture Test Condition	Decline of Absorption Moisture (%)	Ref.
1	2003	1.62%	4%	T = 20 °C, RH = 83%, t = 48 h	64.78%	[30]
2	2004	7%	1%	T = 20 °C, RH = 65%, t = 96 h	80%	[31]
3	2006	2.174%	2.17%	T = 25 °C, RH = 90%, t = 8 h	65.28%	[32]
4	2007	5.27%	-	T = 25 °C, RH = 90%, t = 30 h	69.02%	[33]
5	2013	3.04%	2.5%	T = 30 °C, RH = 90%, t = 24 h	88%	[34]

**Table 2:** Performance of coated AN using the chemical coating method.

No.	Year	Absorption Moisture Rate	Mass Ratio of Coating Layer	Absorption Moisture Test Condition	Decline of Absorption Moisture	Ref.
1	2003	11.4%	-	T = 35 °C, RH = 92%, t = 24 h	38.7%	[37]
2	2005	0.21%	0.3%	T = 35 °C, RH = 55%, t = 24 h	56.25%	[38]
3	2009	10.92%	-	T = 20 °C, RH = 88%, t = 8 h	15.08%	[39]

**Table 3:** Performance of coated AN using the encapsulation coating method.

No.	Year	Absorption Moisture Rate	Mass Ratio of Coating Layer	Absorption Moisture Test Condition	Decline of Absorption Moisture	Ref.
1	2003	9.91%	-	T = 32 °C, RH = 90%, t = 24 h	22.50%	[37]
2	2004	2.8427%	1.5%	T = 30 °C, RH = 94%, t = 8 h	49%	[48]
3	2008	0.623%	-	T = 20 °C, RH = 75%, t = 12 h	88%	[49]
4	2011	0.33%	-	T = 30 °C, RH = 75%, t = 24 h	30.6%	[47]
5	2013	8.78%	0.48%	T = 35 °C, RH = 92%, t = 24 h	28.35%	[50]

**Table 4:** Advantages and disadvantages of coating methods.

Method	Advantages	Disadvantages
Physical coating	(1) Simple. (2) Convenient and easy to manufacture. (3) Improves the stability of the particles. (4) Safety. (5) Enhances the compatibility of particles with other materials.	(1) Uses a large amount of coating agent. (2) Thickness of coating layer not easy to control. (3) Large difference of interfacial tension between the surface coating layer and the polarity of AN.
Chemical coating	(1) Small dosage of coating agent. (2) Strong binding force. (3) The hydrophobic group made the thin layer on the surface prevent hygroscopicity.	(1) The surfactant and coupling agent have low molecular weight. (2) The surfactant has a small solubility in water. (3) Low hygroscopicity properties.
Encapsulation coating	(1) Improves the physical properties of coated particles on the surface. (2) Protects the particles from external moisture. (3) Polymer hygroscopicity was zero. (4) Coating layer is thin.	(1) The brittle polymer is susceptible to cracking during the drying process. (2) There are sticky polymer adhesives not dispersed. (3) Polymer polarity low is difficult to stick on surface of AN particles.
